# Malignant Isolated Cortical Vein Thrombosis as the Initial Manifestation of Primary Antiphospholipid Syndrome: Lessons on Diagnosis and Management From a Case Report

**DOI:** 10.3389/fimmu.2022.882032

**Published:** 2022-04-25

**Authors:** Jie Shen, Zi Tao, Wei Chen, Jing Sun, Yan Li, Fangwang Fu

**Affiliations:** ^1^ Department of Neurology, The Second Affiliated Hospital and Yuying Children’s Hospital of Wenzhou Medical University, Wenzhou, Zhejiang, China; ^2^ Department of Radiology, The Second Affiliated Hospital and Yuying Children’s Hospital of Wenzhou Medical University, Wenzhou, Zhejiang, China

**Keywords:** cortical vein thrombosis, antiphospholipid syndrome, anticoagulation, decompressive craniectomy, magnetic resonance, case report

## Abstract

**Background:**

Antiphospholipid syndrome (APS) with isolated cortical vein thrombosis (ICoVT) is an extremely rare but potentially malignant entity. It is particularly challenging to diagnose APS-related ICoVT because of the non-specific clinical manifestations and the frequent absence of typical neuroimaging. Moreover, there is currently limited knowledge on the clinical features and management strategies for the condition. Delays in diagnosis and treatment may lead to life-threatening consequences.

**Case Presentation:**

We present a rare case of a 74-year-old Chinese woman who presented with sudden onset of headache and right arm weakness that mimicked acute ischemic stroke. Her initial computed tomography was unremarkable, and intravenous thrombolysis was performed. Serial neuroimages confirmed ICoVT 4 days after symptom onset, and low-molecular-weight heparin (LMWH) was started at a dose of 0.4 ml twice per day, according to the 2019 Chinese guidelines. The workup for the predisposing causes of ICoVT revealed triple positivity APS. LMWH dose was adjusted according to the anti-Xa chromogenic assay. However, the patient’s condition deteriorated rapidly, and there was a progressive enlargement of the venous infarction despite treatment with anticoagulants. Transtentorial herniation developed on day 12, and decompressive craniectomy was immediately performed. The patient’s symptoms did not improve significantly after surgery, and she remained aphasic and hemiplegic at the 3-month follow-up, with a modified Rankin Scale score of 5.

**Conclusion:**

ICoVT is a rare yet potentially fatal manifestation of APS, and its diagnosis and treatment are extremely challenging. Timely diagnosis, prompt treatment, and close monitoring are essential to improve the clinical prognosis of patients with APS-related ICoVT.

## Introduction

Isolated cortical vein thrombosis (ICoVT) is a relatively rare and poorly understood phenomenon that represents <0.1% of all stroke entities and 6%–8.7% of all cerebral venous sinus thrombosis (CVST) cases (1, 2). ICoVT is defined as thrombosis that only involves the cortical veins (CoVs) without occlusion of the dural venous sinuses and deep veins (1). The diagnostic challenge for ICoVT is attributed to the non-specific clinical manifestations, frequent anatomical variations in CoVs, and difficulties in determining ICoVT using conventional imaging modalities, such as magnetic resonance imaging (MRI) and magnetic resonance venography (MRV). In contrast to patients with CVST, patients with ICoVT often have parenchymal involvement (81% vs. 40.1%, respectively) and hemorrhagic lesions (46% vs. 13.4%, respectively) (3, 4). Although most ICoVT patients achieve a favorable prognosis, delays in diagnosis and treatment can lead to life-threatening consequences (2, 3). Additionally, it is important to note that CoV involvement is strongly associated with malignant CVST, which is defined as a supratentorial cortical venous infarction alongside malignant brain edema, leading to transtentorial herniation either at onset or after exacerbation (5).

Antiphospholipid syndrome (APS) is a non-inflammatory autoimmune disease characterized by persistent circulating antiphospholipid (aPL) antibodies (6, 7). Common clinical features of APS include venous thromboembolism, stroke, and recurrent abortion (6). APS contributes to 6%–17% of CVST cases, yet it is rarely considered an etiology of ICoVT (3, 8, 9). To date, only three cases of ICoVT have been reported to be associated with APS (10–12). Strikingly, APS-related CVST patients appear to have higher rates of disability and mortality than those of CVST patients without APS, especially APS-related ICoVT patients, in whom one out of the three reported cases experienced malignant cerebral edema and underwent decompressive hemicraniectomy (10).

Here, we present an extremely rare case of a primary APS patient who presented with malignant ICoVT as an initial presentation, whose condition rapidly deteriorated despite anticoagulation treatment. This case highlights our concern among clinicians for this rare yet potentially fatal disease. We discuss the lessons that can be learned from the diagnosis and management of this rare disease and how such lessons can aid in improving patient care and outcomes. Furthermore, we discuss the importance of radiologic monitoring and anti-Xa activity measurement to guide anticoagulant therapy for patients experiencing clinical deterioration.

## Case Presentation

A 74-year-old Chinese woman (gravida 3 para 3) was admitted to the emergency department with sudden onset of headache and right arm weakness for 2.5 h. She had a history of well-controlled hypertension and hyperlipemia, for which she took 5 mg of amlodipine daily and 10 mg of atorvastatin daily as instructed by her primary care provider. She had no family history of thrombotic disease and no history of venous thrombosis. She also denied any history of risk factors for thrombosis (e.g., oral contraceptives, malignancy, and autoimmune diseases).

**Figure 1:**
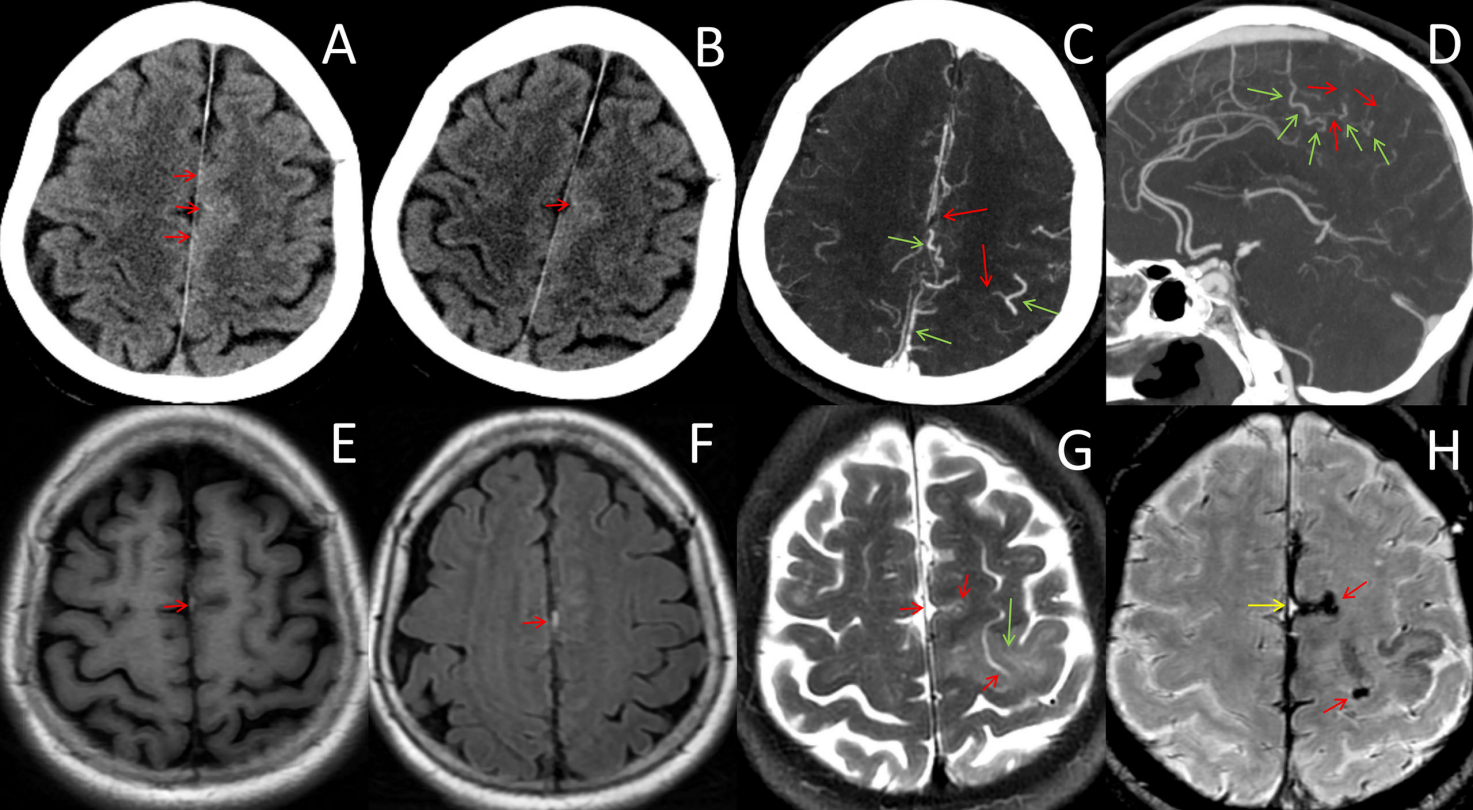
Neuroradiological data from onset to day 4. **(A, B)** CT images before and after intravenous thrombolysis showed no abnormalities except punctate hyperdensities within the left frontal sulci (red arrows). **(C, D)** CT angiography on day 4 showed filling defects of cortical veins (red arrows) and compensatory expansion of the adjacent veins (green arrows). **(E, F)** MRI on day 3 showed hyperintense T1WI and FLAIR lesions neighboring the falx cerebri and the left parietal cortex (red arrows), which were initially diagnosed as subarachnoid hemorrhages, indicating subacute thrombosis in the cortical veins. **(G)** MRI on day 4 showed venous infarction in the frontal lobe (green arrow) and the absence of the flowing-void effect in the cortical veins (red arrows). **(H)** Susceptibility-weighted imaging on day 4 showed curvilinear, serpentine, and exaggerated susceptibility within the sulcus, which was suggestive of thrombosed veins during the acute (red arrows) and subacute phases (greed arrow).

On admission, the patient’s vital signs were within normal limits except for a slightly elevated blood pressure of 141/89 mmHg. Her weight was 63 kg. Neurological examination showed Medical Research Council (MRC) grade 3 for the right arm. The National Institute of Health stroke scale (NIHSS) score at admission was 2. Non-contrast computed tomography (NCCT) was performed, and the radiologist reported no acute abnormalities (**Figure 1A**). The emergent blood assay revealed no abnormalities except for an elevated D-dimer level at 2.4 µg/ml (normal range, 0–0.5 µg/ml). Contraindications for intravenous thrombolysis (IVT) were excluded. After obtaining informed consent from the patient, IVT with 54 mg alteplase was administered 3 h post-onset. The neurological deficits and headache progressively improved, and the NIHSS score 1 h after IVT was 0.

On day 2, the patient complained of headache and weakness of the right extremity (MRC grade 4/5). Her D-dimer level was 4.87 µg/ml. Other laboratory workups were negative and included blood routine tests, C-reactive protein (CRP), erythrocyte sedimentation rate (ESR), liver and renal function, lipids panel, glycated hemoglobin, homocysteine, infection panel, and tumor markers (e.g., carcinoembryonic antigen, alpha-fetoprotein, neuron-specific enolase, and cancer antigen‐125). A repeat CT at 24 h showed no hemorrhage (**Figure 1B**). Aspirin (300 mg/day) and rosuvastatin (20 mg/day) were administered as secondary prevention.

The brain MRI on day 3 revealed punctate abnormalities [hyperintensity on T1-weighted imaging (T1WI) and fluid-attenuated inversion recovery imaging (FLAIR)] that neighbored the cerebral falx and left frontal sulcus (**Figures 1E, F** **)**. However, diffusion restrictions, which are the key parameters for identifying cerebral infarctions, were absent in the parenchyma on diffusion-weighted imaging. After consultation with radiologists, a convexity subarachnoid hemorrhage (cSAH) was considered. Given that the patient was clinically stable, aspirin was continued. CT angiography was performed on day 4 and did not find any aneurysm or arteriopathy. Aneurysmal subarachnoid hemorrhage (SAH) was excluded. However, filling defects of CoVs and compensatory expansion of the adjacent veins were present (**Figures 1C, D**). We then re-evaluated the existing radiographic data and found that punctate hyperdensities within the left frontal sulci, which were ignored on the initial NCCT scans, were consistent with the punctate abnormalities on MRI. The punctate hyperdensities were reduced in the repeated CT acquired 24 h after IVT, which suggested thrombi in the cortical veins. Moreover, lower-extremity intermuscular venous thrombosis was identified by ultrasonography. Thus, ICoVT was highly suspected.

The patient complained of severe headache and progression of right limb weakness (MRC grade 3/5). The MRI on the night of day 4 showed a venous infarction in the frontal lobe (**Figure 1G**). Susceptibility-weighted imaging (SWI) showed curvilinear, serpentine-exaggerated susceptibility within the sulcus, which indicated thrombosed veins in the acute phase (**Figure 1H**). The original MRI abnormality was SWI hyperintensive, which corresponded to subacute vein thrombosis. Therefore, ICoVT was confirmed.

The workup for acquired and inherited hypercoagulability [e.g., antinuclear antibody, antineutrophil cytoplasmic antibodies, rheumatoid factor, procalcitonin, protein C, protein S, antithrombin III, coagulation factor activities panel, thrombophilia gene panel, lupus anticoagulant (LA), anticardiolipin (aCL)], and anti-β2-glycoprotein-I (aβ2GPI) antibodies] was performed on the morning of day 5 before the patient underwent anticoagulation. Additionally, screening for an underlying malignancy was initiated, including chest CT, transthoracic echocardiography (TTE), abdominal ultrasound, and transvaginal ultrasound. However, no evidence of malignancy was found. In the absence of infective symptoms, systemic infection (e.g., infective endocarditis, pulmonary infection, and urinary infection) was excluded according to normal white blood cell count, urine analysis, CRP, ESR, procalcitonin TTE, and chest CT. Laboratory workup reported the next day were positive for LA (dilute Russel viper venom time normalized ratio, 1.78; normal range, 0.7–1.2), aCL IgG (77 IU/ml; normal range, <20 U/ml), aCL IgA (63 IU/ml; normal range, < 20 U/ml), aβ2GPI IgG (89 IU/ml; normal range, <20 U/ml), aβ2GPI IgA (65 IU/ml; normal range, <20 U/ml). Factor VIII, Factor VII, and von Willebrand factors significantly increased by 225% (normal range, 50%–150%), 212% (normal range, 50%–150%), and 160% (normal range, 50%–150%), respectively.

**Figure 2:**
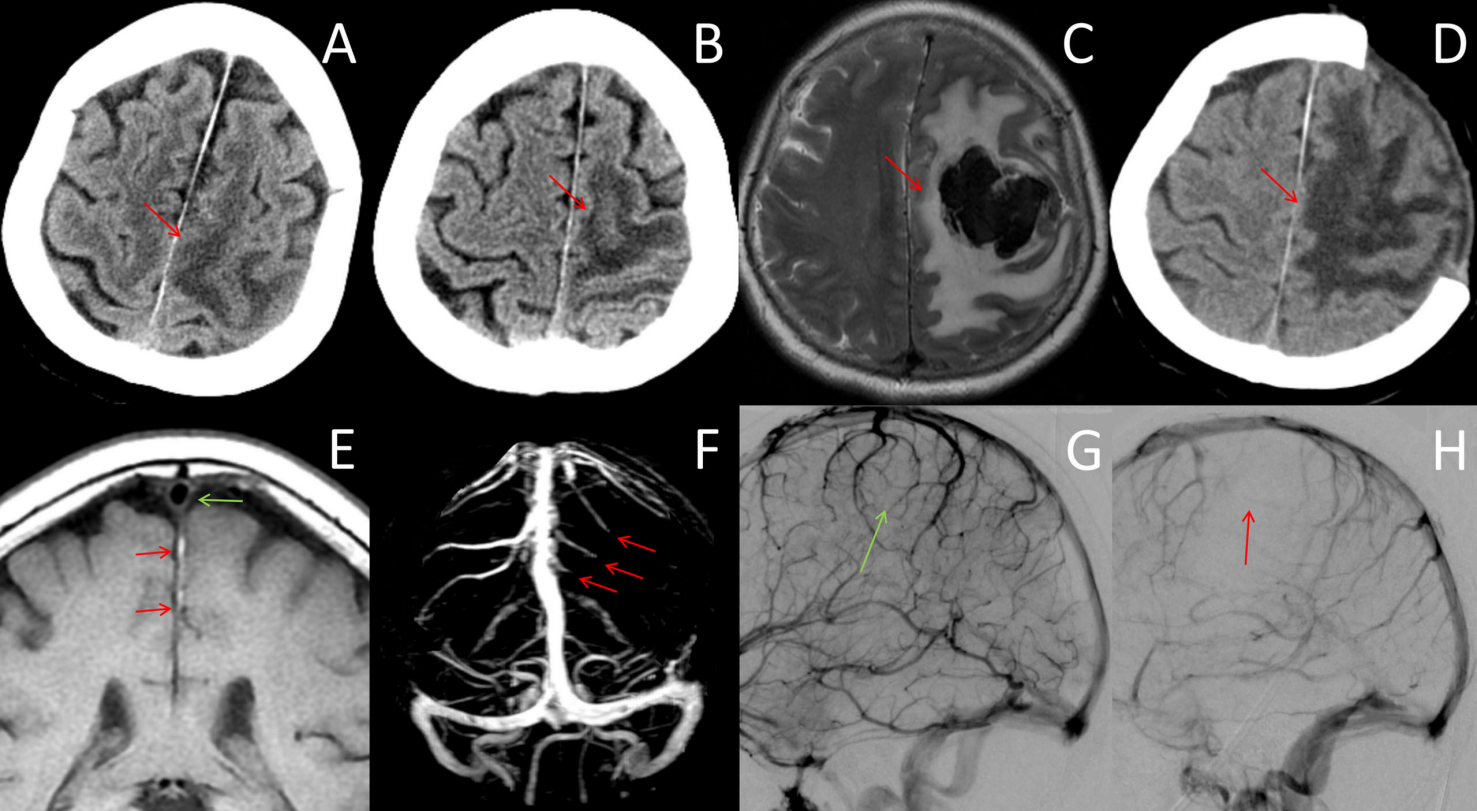
Neuroradiological data after day 4. **(A, B)** CT images on days 6 and 10 showed progressive enlargement of the venous infarction lesion in the frontoparietal cortex (red arrows). **(C)** MRI on day 12 showed venous infarction lesions with a large acute hematoma in the left frontoparietal lobe, which resulted in transtentorial herniation (red arrows). **(D)** CT scan after decompression craniectomy showed large areas of low-density lesions (red arrow). **(E)** MR black-blood-thrombus imaging (MRBTI) on day 7 showed vein thrombosis that extended proximally to the superior sagittal sinus (red arrow). The superior sagittal sinus was free of thrombosis (green arrow). **(F)** MR venography showed filling defects of the left frontal and partial cortical veins (red arrows). **(G, H)** DSA on day 8 showed normal venous flow of the right hemisphere (**G**, green arrow) compared with multiple thromboses in the left superior and internal cerebral veins of the left hemisphere (**H**, red arrows).

Aspirin was stopped on the morning of day 5, and low-molecular-weight heparin (LMWH) was started at a dose of 0.4 ml every 12 h according to the 2019 Chinese guidelines for clinical management of CVST (13). On the dawn of day 6, the patient experienced an episode of generalized tonic–clonic seizures, which was controlled with diazepam and levetiracetam (0.5 g twice per day). Electroencephalogram showed epileptiform discharges in the left frontal lobe. An enlargement in the venous infarction was observed on the re-examination CT (**Figure 2A**). According to the institutional periprocedural protocol, lumbar puncture was scheduled 24 h after the final administration of LMWH. The cerebrospinal fluid pressure was 160 mmH_2_O, and cerebrospinal fluid analyses were all within the normal range. Thus, intracranial infection, SAH, and intracranial hypertension were subsequently excluded. LMWH was restarted 4 h after the lumbar puncture.

MR black-blood-thrombus imaging (MRBTI) on day 7 showed vein thrombosis that extended proximally to the superior sagittal sinus (**Figure 2E**). MR venography showed filling defects of the left frontal and partial cortical veins (**Figure 2F**). The patient’s condition deteriorated rapidly, and she developed drowsiness and lethargy; moreover, the muscle strength of her right limbs dropped to grade 1. We employed a multidisciplinary approach combining neurointervention, rheumatology, and hematology consultations. The diagnosis of ICoVT and possible APS was made, and hydroxychloroquine was additionally prescribed. LMWH was recommended to be adjusted with an anti-Xa chromogenic assay. Digital subtraction angiography (DSA) was scheduled to evaluate whether direct intrasinus thrombolysis should be performed.

On day 8, peak anti-Xa was 0.4 IU/ml, and LMWH was increased to 0.6 ml twice per day. DSA demonstrated multiple thromboses of superior and internal cerebral veins without sinus thrombosis (**Figures 2G, H**). However, thrombolysis was negated by the neurointerventionalist. The conditions continued to worsen progressively, with enlargement of the venous infarction in the CT on day 10 (**Figure 2B**). Peak anti-Xa was 0.71 IU/ml, and a high-intensity LMWH dose of 0.8 ml twice per day was taken with a goal of achieving 0.8–1.0 IU/ml.

**Figure 3:**
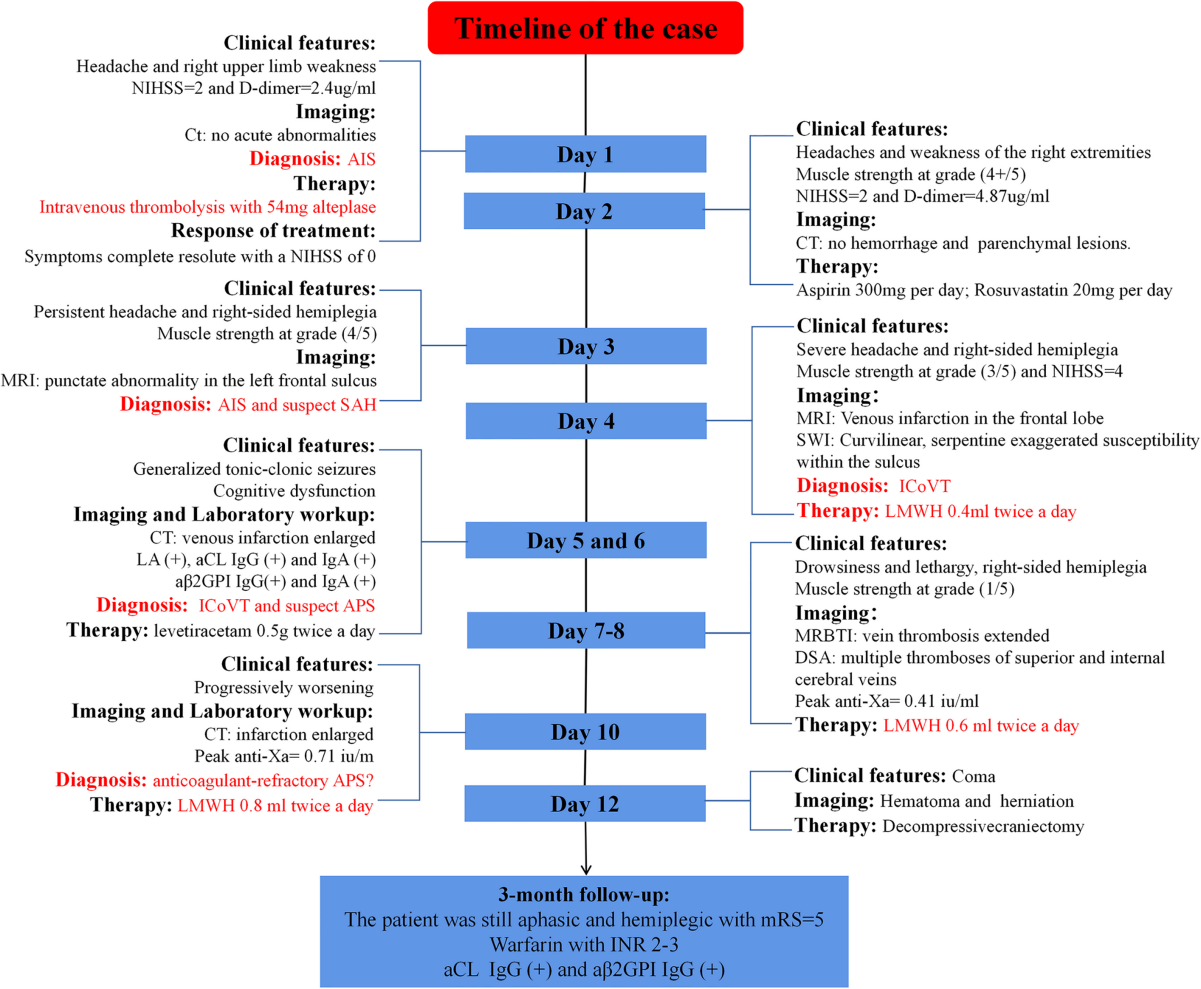
Timeline of the present case.

On day 12, the patient progressed to a coma, and the MRI showed a large intracranial hemorrhage (ICH) in the left frontoparietal lobe and transtentorial herniation (**Figure 2C**). Decompressive craniectomy was performed immediately (**Figure 2D**). LMWH was restarted 3 days after the operation at a dose of 0.4 ml twice per day based on the risk of hemorrhage. Warfarin was given 3 weeks after craniectomy. The patient’s symptoms did not improve significantly after surgery and was thus transferred to a rehabilitation hospital. At the 3-month follow-up, serum aCL IgG (51 IU/ml) and aβ2GPI IgG (71 IU/ml) were positive, and a diagnosis of primary APS was established. LA was not retested because of long-term warfarin therapy. The patient remained aphasic and hemiplegic. Her modified Rankin Scale (mRS) score was 5. The timeline of the case is showed in the **Figure 3**.

## Discussion

It is well-established that ICoVT is the rarest subtype of CVST that presents as an isolated occlusion of anastomotic surface veins (2, 14). It remains a poorly understood condition without established guidelines for rapid identification or precise treatment. Its propensity to mimic not only symptoms but also neuroradiological manifestations of numerous other diseases makes diagnosis particularly challenging (14). Misdiagnoses include acute ischemic stroke (AIS), cSAH, glioma, cerebral parasitic disease, and even multifocal leukoencephalopathy (15). Symptoms and signs such as seizures and motor and sensory disturbances are common in ICoVT, especially in the early stages, which results in the misdiagnosis of the underlying ICoVT (16). As observed in our patient, the sudden onset of right arm weakness without the presence of ICH was consistent with a diagnosis of AIS. Owing to the narrow time window of IVT, differential diagnosis can be challenging. The European Stroke Organization (ESO) guidelines for CVST recommend that D-dimer should be measured before neuroimaging in patients with suspected CVST. Meng and colleagues enrolled 233 patients with an unexplained headache who visited the emergency room. The average D-dimer level was significantly higher in CVT patients (987.7 ± 324 mg/L) than in patients in the mimic group (343.23 ± 102 mg/L), resulting in high sensitivity (94.1%) and specificity (97.5%). Although ICoVT is limited to focal segments of surface veins, D-dimer has also been shown to be sensitive (16). The marked increase in D-dimer level in our case may provide a clue for the diagnosis of ICoVT. The suspicion of venous thrombosis should be raised because of the early onset of headache and the marked increase in D-dimer level, given that these features are rarely seen in patients with lacunar stroke (17). The accuracy of D-dimer for diagnosing acute lacunar stroke is well established, with reported rates of 88% with D-dimer alone and 98% with the conjunctive use of D-dimer and the Oxfordshire Community Stroke Project classification (18). However, elevation of D-dimer is observed in most thrombotic vascular diseases. Moreover, it should be noted that D-dimer levels may rise in certain pathological states besides thrombosis, such as cancer, infection, and liver disease. D-Dimer may also be influenced by various factors, such as age, race, sex, and medications (19). Therefore, D-dimer should be used as part of a comprehensive diagnostic tool for CVST but not as a stand-alone test. Because of the low cost, convenience, and speed of D-dimer measurement, we still recommend to measure D-dimer when suspecting CVST. A high level of clinical suspicion is required to consider ICoVT in patients who present with increased D-dimer level and symptoms of focal neurological deficit and seizures, particularly in young patients and pregnant/puerperal women.

It is not surprising that ICoVT shares similar sex and age distribution, predisposing factors, and clinical presentations as CVST (2, 14). Although female sex predilection and young individuals are well-established factors (2, 14), there are some differences in predisposing factors between ICoVT and CVST. Murumkar and colleagues recently conducted the largest retrospective observational study to date of ICoVT involving 28 patients. They concluded that iron deficiency anemia, hyperhomocysteinemia, infection, and female hormonal imbalance were the top 4 risk factors (2). Systemic autoimmune diseases were rarely considered the cause of ICoVT in their cohort, and only one patient with mixed connective tissue disease was reported. This phenomenon is in line with a previous case series report and a systematic review (3, 20). Song and colleagues reported a similar phenomenon in patients with acute cortical vein thrombosis. Only four (17.4%) patients had underlying autoimmune diseases (16), two of whom had APS but not ICoVT and were identified as having abnormalities in the cerebral sinus using MRBTI. Despite the scarcity of publications, physicians should certainly not neglect APS as a trigger factor for ICoVT given the potential malignant nature of APS-related ICoVT.

APS is an immune-mediated thrombophilic disorder propelled by a heterogeneous group of persistent circulating aPLs and associated proteins (9, 21). APS is characterized by recurrent vascular thrombosis, pregnancy morbidity, and non-criteria manifestations, such as livedo reticularis, thrombocytopenia, and valve disease (6, 9). Well‐established antiphospholipid antibodies include aCL, aβ2GPI, and LA. The diagnosis of APS still relies on the 2006 Sydney criteria (6). As the clinical events of thrombosis can occur in the vascular beds of any size and organ, the clinical presentation of APS varies enormously (9). APS contributes to 6%–17% of CVST patients across cohort studies (9). However, CVST is a relatively rare presentation of APS, with a reported prevalence of only 0.7% (9, 22). A recent systematic review on APS-related CVST patients showed that CVST predominantly affects young women and involves the superior sagittal sinus and lateral venous system (60% and 69%, respectively) (23). Extensive thrombosis is a vital characteristic of APS-related CVST patients, with an incidence of 60% (23). Only 1.8% of APS-related CVST are identified as ICoVT (23). Thus, it is not difficult to understand that ICoVT is an extremely rare presentation of APS that depends on the propensity of APS to develop into widespread vascular thrombosis. To the best of our knowledge, ICoVT has occurred in only four patients with APS to date, including our case (10–12). The detailed information of the cases is listed in the **Table 1**. It is vital to highlight that half of these patients presented with malignant ICoVT despite undergoing anticoagulation. In addition, the mortality of APS-related CVST is likely to be higher (6.4%–15.8%) than those without APS (<5% in most studies) (23–25). This suggests that patients with APS are more likely to have a poor outcome and require close monitoring, especially high-risk patients with high adjusted global APS (aGAPSS) scores (26).

**Table 1:** Clinical and neuroradiological characteristics of APS-related ICoVT.

	Present case	Polster et al. (10)	Numata et al. (11)	Miranda et al. (12)
**Age and sex**	73/Female	16/Female	45/Male	29/Female
**Risk factors**	Hypertension, hyperlipemia	Post-partum	Tadalafil ingestion	OC, smoking
**Clinical course**	Acute	Acute	Acute	Acute
**Initial symptoms**	Headache, focal neurological deficit	Focal neurological deficit	Posterior headache	Focal neurological deficit focal epilepsy and GTCS
**Initial diagnosis**	Acute ischemic stroke	Acute stroke	Tension headache or cerebrospinal fluid hypovolemia	Not mentioned
**Sequential symptoms**	GTCS, consciousness disturbance	GTCS, consciousness disturbance	GTCS, consciousness disturbance	GTCS
**Involved veins**	Left superior and internal cerebral veins	Left superior cerebral veins	Right parietal CoV	Left frontal CoV
**Parenchymal changes**	VI in frontoparietal cortex, then converted to hemorrhagic VI	Hemorrhagic VI in frontoparietal cortex	VI in right parietal cortex	VI in left prefrontal cortex
**Neuroradiological signs of ICoVT**	CT: punctate hyperdensities in the sulci MRI: subacute CoV thrombus SWI: exaggerated susceptibility within the sulcus	CT: cord sign CTV: filling defect of CoV	CT: CT cord sign SWI: SWI cord sign	GRE T2WI: cord sign
**DSA**	Filling defect of CoVs	Not performed	Not performed	Not performed
**APS Criteria**	Sydney	Sapporo	Sydney	Not mentioned
**APS Antibodies**	LA, aCL, IgG, and IgA, aβ2GPI IgG and IgA	LA	aCL, IgG, and aβ2GPI IgG	aCL IgM
**Laboratory abnormalities**	Elevated D-dimer	No	Elevated D-dimer	No
**Anticoagulation**	Low-molecular-weight heparin, then warfarin	Heparin, then warfarin	Heparin, then warfarin	Heparin, then warfarin
**Complications**	Hemorrhage, brain herniation	DIC, brain herniation	No	No
**Decompression craniectomy**	Yes	Yes	Not performed	Not performed
**Recurrence**	No	No	No	No
**Prognosis**	Poor, mRS score of 5 at 3 months	Poor, mRS score of 4 at 6 months	Excellent	Excellent

aβ2GPI, anti-β2-glycoprotein-I antibody; aCL, anticardiolipin antibody; CoV, cortical vein; CT, computed tomography; CTV, computed tomography venography; GRE, gradient-echo images; GTCS, generalized tonic–clonic seizures; LA, lupus anticoagulant; MRI, magnetic resonance imaging; mRS, modified Rankin Scale; OC, oral contraceptives; VI, venous infarction.

Because of the non-specific clinical manifestations of ICoVT, neuroimaging is the cornerstone of ICoVT diagnosis (2, 27). However, precise imaging diagnosis is challenging owing to the nature of CoVs, which include their relatively small size, anatomical variations, and collateral circulation. There are many lessons to be learned from our case in regard to neuroimaging diagnosis. First, the punctate hyperdensities within the left frontal sulci were ignored on the initial NCCT scans. NCCT is usually the first diagnostic tool used, especially for patients in the emergency department. The CT “cord sign” is the direct visualization of the thrombosed cortical veins, which present as cord-like hyperdensities in the cerebral sulcus (15, 28). This sign is rare and has low diagnostic sensitivity for ICoVT. Indeed, in a cohort of 71 patients, no patient presented this sign (28). We speculate that punctate hyperdensities in the sulci are equivalent to the cord sign as a result of the limited amount of thrombosis in cortical veins. Other indirect signs of NCCT, such as narrowing of the neighboring sulci and sulcal hemorrhage, are also uncommon (28). Therefore, ICoVT is easily overlooked on NCCT, and patients with predisposing risk factors or an increased D-dimer level require further workup despite normal NCCT. Interestingly, we observed an improvement in the punctate hyperdensities on NCCT after IVT, which suggested partial dissolution of the thrombus. Second, ICoVT mimics cSAH on MRI, which led to a delay in diagnosis and treatment in our case. The cSAH has been reported as a complication of IVT; thus, it must be considered a differential diagnosis of post-IVT headache. The repeat CT showed improvement in punctate hyperdensities, which made the diagnosis of cSAH less likely. Subacute ICoVT (5–14 days) is difficult to differentiate from cSAH because of the similar radiographic appearance when parenchymal involvement is absent (29). Subacute ICoVT and cSAH are usually observed along the sulci and are shown as hyperdensities on NCCT and hyperintensities on FLAIR images. On conventional MRI, acute ICoVT is isointense on T1WI, hypointense on T2WI and SWI, whereas subacute ICoVT is hyperintense on T1WI and T2WI and is also called the hyperintensive vein sign. SWI can help with the differential diagnosis of SAH. Third, vascular lesions should be carefully evaluated, rather than focusing on parenchymal lesions. Changes in parenchymal lesions always lag behind the development of the underlying venous thrombosis. Although the venous infarction was localized to the left parietal cortex, DSA revealed multiple thromboses of CoVs in our case, which resulted in malignant complications. These imaging findings should be considered as warning signs of clinical deterioration. Given that supratentorial cortical thrombosis is more vulnerable to transtentorial herniation, more aggressive therapy may be required to prevent disease progression (30). Careful imaging analysis is particularly critical for clinical decision-making.

The diagnosis of ICoVT remains relatively difficult despite progress in imaging techniques (2). Non-contrast MRI may help detect thrombosis in small peripheral cortical veins based on these signs but only during the acute to subacute phases (27). In our patient, the MRI acquired on day 3 showed hyperintense T1WI, T2WI, and FLAIR lesions neighboring the falx cerebri and the left parietal cortex, which suggested subacute thrombosis in the CoVs. The small filling defect of the peripheral CoVs on contrast CT enabled us to confirm the presence of thrombosis; however, it could have been easily missed by the radiologists. Contrast CT is reported to have low sensitivity for small vein occlusions and is rarely used in clinical practice (2). Post-contrast TIWI demonstrated fair sensitivity to contrast CT in a previous study, with only limited value in the detection of thrombosis in the venous sinus and large CoVs (2). Previously, DSA was the gold diagnosis standard for ICoVT owing to its ability to detect slow blood flow and filling defects in CoVs. However, its diagnostic value in ICoVT is somewhat questionable, with a reported diagnostic rate of approximately 14.3%–47% (16). Contrast-enhanced MRV has been reported to be the best routine diagnostic tool for ICoVT, with a diagnostic rate of 45%–73% (3, 14). Despite the shortcomings in demonstrating distal cortical vein thrombosis and distinguishing anatomic venous variations, contrast-enhanced MRV is a fast, non-invasive, and recommended technique. SWI can detect different forms of iron in the tissue and is effective at visualizing CoV thrombosis and venous stasis surrounding the thrombosis (2, 14). However, its pitfalls near bone interfaces and prior hemorrhage should be excluded carefully (2). Recent advances in MRBTI have enabled the qualitative evaluation of the pathological stages of the venous thrombus and the quantitative assessment of the thrombus load (2, 16). MRBTI has excellent value in identifying cortical vein thrombus, with a sensitivity rate of 100%; moreover, it is highly efficient at distinguishing ICoVT from CoVT comorbid with CVST (16).

LMWH or unfractionated heparin followed by oral antagonists for an individualized duration according to predisposing causes is recommended by the current clinical guidelines (9, 13). However, the therapy strategy, including drug choice, dose intensity, and treatment duration, remains controversial in the context of different comorbid diseases. For example, thrombocytopenia is a common yet crucial complication of APS, which raises a dilemma for the management of APS-related CVST (7, 9). Furthermore, there are several dilemmas for APS-related ICoVT. First, ICH is particularly common in APS-related ICoVT, which raises the question of when to start and how to manage anticoagulation in the presence of ICH. The 2019 Chinese guidelines and the ESO guidelines state that minor ICH is not an absolute contradiction to anticoagulation. The evidence for this recommendation is derived from two randomized control trials that reported mean delays in anticoagulation treatment of 10.9 and 32.5 days after initial symptom onset, although they only included patients with minor ICHs who had been stable for several days (10). Thus, there is currently a lack of evidence on the optimum timing for starting anticoagulation. The early initiation of anticoagulation in patients with hemorrhagic CVST has been called into question, especially those with a large or expanding ICH. Previous studies have reported that the presence of early ICH is strongly associated with expanding or new-onset ICH following intravenous unfractionated heparin treatment (23.7% vs. 10.9%) (31). Considering the possibility of further aggravation of the ICH, hemorrhagic CVST remains a therapeutic challenge. Second, CoV thrombosis tends to be malignant and life threatening despite anticoagulation. Therefore, understanding how to monitor and manage anticoagulation is crucial. Third, how should anticoagulant-refractory ICoVT, as observed in our patient, be managed? The evidence and guidelines on these conditions are currently lacking.

Much can be learned from our failed treatments. For example, the aGAPSS can help identify patients at a high risk of thrombosis development and recurrence (7, 9). In the 2019 Chinese guideline for CVST, the therapeutic dosage of LMWH was 0.4–0.6 ml twice per day (13). However, the recommended dosage may not be sufficient for patients with APS and should be adjusted according to risk stratification and anticoagulation monitoring (7, 26). The aGAPSS, which is developed to quantify thrombosis risk, is calculated by adding together the corresponding points to risk factors: 5 for aCL (IgG/IgM), 4 for LA, 4 for aβ2GPI (IgG/IgM), 3 for dyslipidemia, and 1 for arterial hypertension (32). aGAPSS risk is stratified as high risk (≥ 12 points), medium risk (6–11 points), and low risk (<6 points) according to Radin’s classification (33). Our case had triple positivity and an aGAPSS of 17 points, which resulted in the highest risk for venous thrombosis; moreover, she presented with gradual deterioration of neurological function despite receiving standard-dose LMWH. A reasonable approach is high-intensity LMWH at approximately 25% or 33% above the standard dose. The anti-Xa chromogenic assay is the best assay for monitoring LMWH (23); the recommended peak anti-Xa is 0.5–0.8 IU/ml for standard treatment and 0.8–1.0 IU/ml for high-intensity treatment (7, 23). In fact, both the initial and adjusted LMWH doses in our case appeared insufficient for achieving our treatment goals. Thus, we recommend that the LMWH dose be adjusted according to the anti-Xa chromogenic assay, especially in anticoagulant-refractory patients. Additionally, MRBTI can non-invasively and quantitatively assess the thrombus load, which is of significant value in the evaluation of anticoagulation effects. However, catastrophic APS requires careful consideration when thromboses occur in multiple organs. Aside from providing adequate anticoagulation and eliminating precipitating factors, early treatment is critical for catastrophic APS and includes corticosteroids, intravenous immunoglobulin, plasma exchange, B-cells inhibition, and complement inhibition (34). Awareness that direct-acting oral anticoagulants should be avoided in patients with APS is critical (9, 23). Vitamin K antagonist with a target INR of 2–3 is strongly recommended for long-term prevention (2, 13).

Although anticoagulation therapy remains the cornerstone of treatment to prevent thrombosis in primary APS patients, this treatment may be insufficient in more than 5% of cases (35, 36). Hydroxychloroquine, which was used in our case, has gradually become recognized as an effective adjuvant treatment for thrombosis in APS patients owing to its antithrombotic, anti-inflammatory, and immunomodulatory properties (35, 36). In a recent randomized prospective study involving 50 patients with primary APS, patients treated with hydroxychloroquine had a 91% lower risk of thrombosis than patients who received standard care alone (hazard ratio, 0.09; 95% confidence interval, 0.01–1.26, *p* = 0.074) (35). In addition, the administration of hydroxychloroquine can significantly reduce thrombus size and duration (36).

Several therapeutic options are available for APS-CVST patients who are refractory to anticoagulant therapy. Current evidence does not support the use of systemic thrombolysis before anticoagulant therapy. Ongoing anticoagulant therapy is a contraindication of systemic thrombolysis. Recent years have seen an increase in the use of endovascular therapy (EVT), including intrasinus thrombolysis (IST) and mechanical thrombectomy (MT), in patients who are refractory to anticoagulant therapy (37). Thrombolytic agents can be directly delivered to the site of the thrombosed venous to dissolve the thrombus. One study of 156 CVST patients treated with IST showed that functional independence was achieved in 91.38% of patients, whereas complete or partial recanalization was achieved in 93.96% of patients (38). The rate of major hemorrhagic complications has been reported to be 9.8%–10.7% across studies (37). MT is an alternative EVT method that offers higher rates of successful recanalization and a lower risk of hemorrhagic complications. Various techniques and devices have been applied to clinical practice with and without IST. A systematic review of 17 studies and 235 severe CVST patients showed that a favorable outcome is achieved in 76% of patients, and the mortality rate is 14.3% (39). Notably, 87.6% of patients underwent concomitant IST and MT (39). As mentioned above, IST and MT may be reasonably effective and safe in patients with a high risk of poor prognosis. Recently, a retrospective study reported that IST and MT are also effective and safe in most patients with CVST with ICH (40), which strongly reinforces the hypothesis that ICH is not a contraindication for IST or MT. However, these therapeutic options may not be applicable to ICoVT patients because of the distinctive histopathological characteristics of ICoVT.

Decompressive craniectomy (DC) is a well-established life-saving procedure and also the only treatment for malignant CVST or ICoVT (41). Because evidence regarding the benefits of DC was limited until 2011, the 2011 American Heart Association/American Stroke Association guideline only weakly recommended that DC “might be” needed as a life-saving measure (42). Recently, growing evidence indicates that DC not only improves survival but also achieves acceptable outcomes, even in critically ill patients. A systematic review of five studies published from 2012 to 2018 evaluated the role of DC in CVST. In the pooled analysis, the mortality rate was 16.1%, and favorable outcomes with an mRS score of 0–2 were achieved in 54.4% of patients (41). Thus, the ESO guidelines and the 2019 Chinese guidelines strongly recommend DC for patients with impending herniation (13, 43). Additionally, a recent retrospective study of 48 patients with malignant CVST who were divided into a DC group and a medical group (44) reported that the mortality and favorable outcome rates of the DC group were consistent with those of previous studies. However, none of the patients in the medical group survived during hospitalization, which indicates that patients with malignant CVST do not benefit from endovascular or medical treatment (44). The current guidelines do not elaborate on the indications for DC (13, 43). In the published literature, DC is recommended to be offered as early as possible in the presence of clinical or radiological signs of impending or established herniation, such as third nerve palsy, progressive neurological deterioration with a decrease in Glasgow coma score of more than 4 points, the threat of cerebral herniation, midline shift of ≥ 5 mm, and obliteration of the basal cisterns (41, 44). The optimal timing for restarting anticoagulation has not yet been elucidated.

## Conclusion

ICoVT is a rare yet potentially fatal manifestation of APS. Clinicians should consider ICoVT in patients who present with an increased D-dimer level and symptoms of focal neurologic deficit and/or seizures. APS should be considered in patients with ICoVT, particularly young women. Neuroimaging is the cornerstone of ICoVT diagnosis, and multi-parametric MRI is required to identify this rare entity. Prompt anticoagulation and close monitoring are essential to achieve good clinical outcomes in patients with APS-related ICoVT.

## Data Availability Statement

The original contributions presented in the study are included in the article. Deidentified data, including clinical manifestations, neuroimaging data, serum tests, and cerebrospinal fluid tests, are available upon appropriate request to the corresponding author.

## Ethics Statement

Written informed consent was obtained from the individual(s) for the publication of any potentially identifiable images or data included in this article.

## Author Contributions

FF and YL contributed to the design of the study, revised the manuscript, and were responsible for the integrity and accuracy of the data. JShen contributed to drafting the manuscript and reviewing the published literature. ZT contributed to the collection of clinical and neuroimaging data. JSun was the attending doctor of the patient and contributed to the acquisition and analysis of the clinical data. WC contributed to the analysis of the neuroimaging data. All authors contributed to the article and approved the submitted version.

## Funding

This work was supported by the Medical Science and Technology Project of Zhejiang Province of China (No. 2021KY797), the Natural Science Foundation of Zhejiang Province of China (No. LY21H090015), and the Wenzhou Basic Scientific Research Project (No. Y20210900).

## Conflict of Interest

The authors declare that the research was conducted in the absence of any commercial or financial relationships that could be construed as a potential conflict of interest.

## Publisher’s Note

All claims expressed in this article are solely those of the authors and do not necessarily represent those of their affiliated organizations, or those of the publisher, the editors and the reviewers. Any product that may be evaluated in this article, or claim that may be made by its manufacturer, is not guaranteed or endorsed by the publisher.
